# Cost- effectiveness of HPV vaccination regime: comparing twice versus thrice vaccinations dose regime among adolescent girls in Malaysia

**DOI:** 10.1186/s12889-016-2754-1

**Published:** 2016-01-23

**Authors:** Syed Aljunid, Nimetcan Mehmet Orhun, Amrizal M Nur, Mohd Rushdan Md Noor, Sharifa Ezat Wan Puteh

**Affiliations:** 1 International Centre for Casemix and Clinical Coding, Faculty of Medicine, Jalan Yaacob Latiff, Kuala Lumpur, 56000 Malaysia; 2 Department of Health Management, Faculty of Health Science, Necmettin Erbakan University, Konya, Turkey; 3 Department of Obstetrics and Gynaecology, Hospital Sultanah Bahiyah Alor Setar Kedah, Alor Setar, Malaysia; 4 Department of Community Health, UKM Medical Center, Kuala Lumpur, Malaysia; 5 Department of Health Policy and Management, Faculty of Public Health, Kuwait University, P.O Box 24923, 13110 Safat, Kuwait

**Keywords:** HPV, Vaccine, 3-doses, 2- doses, Schoolgirls, Malaysian girls

## Abstract

**Background:**

The HPV vaccine was introduced to Malaysian national immunization programme in 2010. The current implementation age of HPV vaccination in Malaysian is at the age of 13 years school girls, given according to a 3 doses protocol which may complicate implementation and compliance. Aim of the study is to determine the cost-effectiveness of HPV vaccination regime comparing twice versus thrice HPV vaccinations dose regime among adolescent girls in Malaysia.

**Methods:**

A Markov cohort model reflecting the natural history of HPV infection accounting for oncogenic and low-risk HPV was adapted for 13 year old Malaysian girls cohort (*n* = 274,050). Transition probabilities, utilities values, epidemiological and cost data were sourced from published literature and local data. Vaccine effectiveness was based on overall efficacy reported from 3-doses clinical trials, with the assumption that the 2-doses is non-inferior to the 3-doses allowing overall efficacy to be inferred from the 3-doses immunogenicity data. Price parity and life-long protection were assumed. The payer perspective was adopted, with appropriate discounting for costs (3 %) and outcomes (3 %). One way sensitivity analysis was conducted. The sensitivity analysis on cost of vaccine, vaccine coverage and discount rate with a 2-doses protocol was performed.

**Result:**

The 3-doses and 2-doses regimes showed same number of Cervical Cancers averted (361 cases); QALYs saved at 7,732,266. However, the lifetime protection under the 2-doses regime, showed a significant cost-savings of RM 36, 722,700 compared to the 3-doses scheme. The MOH Malaysia could vaccinate 137,025 more girls in this country using saving 2-doses regime vaccination programme. The model predicted that 2-doses HPV vaccination schemes can avoid additional 180 Cervical Cancers and 63 deaths compare to 3-doses.

**Conclusion:**

A 2-doses HPV vaccination scheme may enable Malaysian women to be protected at a lower cost than that achievable under a 3-doses scheme, while avoiding the same number of Cervical Cancer cases and deaths. Using the saving money with 2-doses, more Cervical Cancers and deaths can be avoided.

## Background

World Health Organization (WHO) has recommended that routine human papillomavirus (HPV) vaccination for 9–13 year old girls be included in national immunization programmes in countries where: 1) the prevention of cervical cancer and/or other HPV-related diseases is a public health priority, 2) vaccine introduction is programmatically feasible, 3) sustainable financing can be secured, and 4) the cost-effectiveness of vaccination strategies in the country or region has been duly considered since 2009 [1].

By the end of 2011, 40 countries had introduced HPV vaccine in their national immunization schedule [2]. Global experience with HPV vaccine delivery to its target population of 9 – 13 year old girls remains limited, particularly in resource-poor settings. Furthermore, there are many stakeholders and partners for HPV vaccine introduction and cervical cancer prevention who are new to immunization and are not the traditional child health partners of immunization programmes, but who bring experience from the fields of reproductive health, adolescent health, school health, cancer control, HIV prevention, and women’s health. In this complex context, there is a need to optimally coordinate the energy, advocacy, and resources of the many stakeholders and partners so that critical vaccine delivery issues are addressed and so that countries can best benefit from the new opportunity that HPV vaccine can offer [3].

The recommended target population for HPV vaccine is 9 to 13 year old girls, a population that has not been routinely served by immunization programmes in most low or low middle income countries (LMICs). Thus, a decision to introduce HPV vaccine in such countries requires creation of new vaccine delivery services in order to deliver 3 doses to each girl. Unlike new infant vaccines which may be added to an existing infant vaccine delivery system, 9–13 year old children in many parts of the world currently receive limited or no routine preventive or other health services, so there is also limited or no existing preventive health service delivery system in place on which HPV vaccine delivery can depend. However, in some LMICs, HPV vaccination will be easier to introduce since school health programs are already in place in many countries and are already giving booster vaccinations. Thus, before introducing HPV vaccine, policymakers and program managers must understand the costs both of procuring the vaccine and of delivering the vaccine [4].

Recent clinical trial studies demonstrated that two doses of HPV vaccine could be as protective as three doses in the short-term. A nested non randomized analysis within a phase III randomized clinical trial in Costa Rica demonstrated that two doses of HPV vaccine has similar high efficacy against vaccine-type persistent infections as three doses, four years after vaccination [5]. Recent study, a phase III randomized trial examined the immunogenicity of two doses in girls 9–13 years compared to three doses in girls 9–13 years and three doses among young women 16–26 years. Results from the study showed that antibody responses for the vaccine-types among girls (9–13 years) who received two doses were no inferior to those among young women (16–26 years) who received three doses, over a period of three years after the last vaccine dose [6]. More data on duration of protection is required before reduced-dose schedules are recommended or implemented. However, such information will not be available for several years. Furthermore, data on duration of protection is not typically avail- able when new vaccines are introduced such as duration of three-dose HPV vaccine protection is still unknown [7].

Malaysia is one of the countries that introduced the HPV vaccine in to her national immunization programme in 2010, targeting schoolgirls [8]. The current implementation age of HPV vaccination in Malaysian is at the age of 13 years girls, given according to a 3-dose protocol that may complicate implementation and compliance. Aim of this study is to determine the cost-effectiveness of HPV vaccination regime comparing twice versus thrice HPV vaccinations dose regime among adolescent girls in Malaysia.

## Methods

### Study design

This is a cost-effectiveness study, to estimate the economic burden of different doses regime of HPV vaccinations in preventing cervical cancers (CC) and to measure cost- effectiveness of these two options to prevent cancer of cervix. Additionally, 2 does regime versus no vaccination analysis was run. Patients are not interviewed for this study as data for QOL and QALYs are from published secondary data. Costing data for this study was obtained from two public hospitals, the Hospital Universiti Kebangsaan Malaysia and Hospital Sultanah Bahiyah, Alor Setar, Kedah from October 2012 to November 2013 by using standard costing template developed by researchers. Cost estimation was carried out via two different components, namely; Step-down costing and expert’s opinion.

### Model structure

**Fig. 1:**
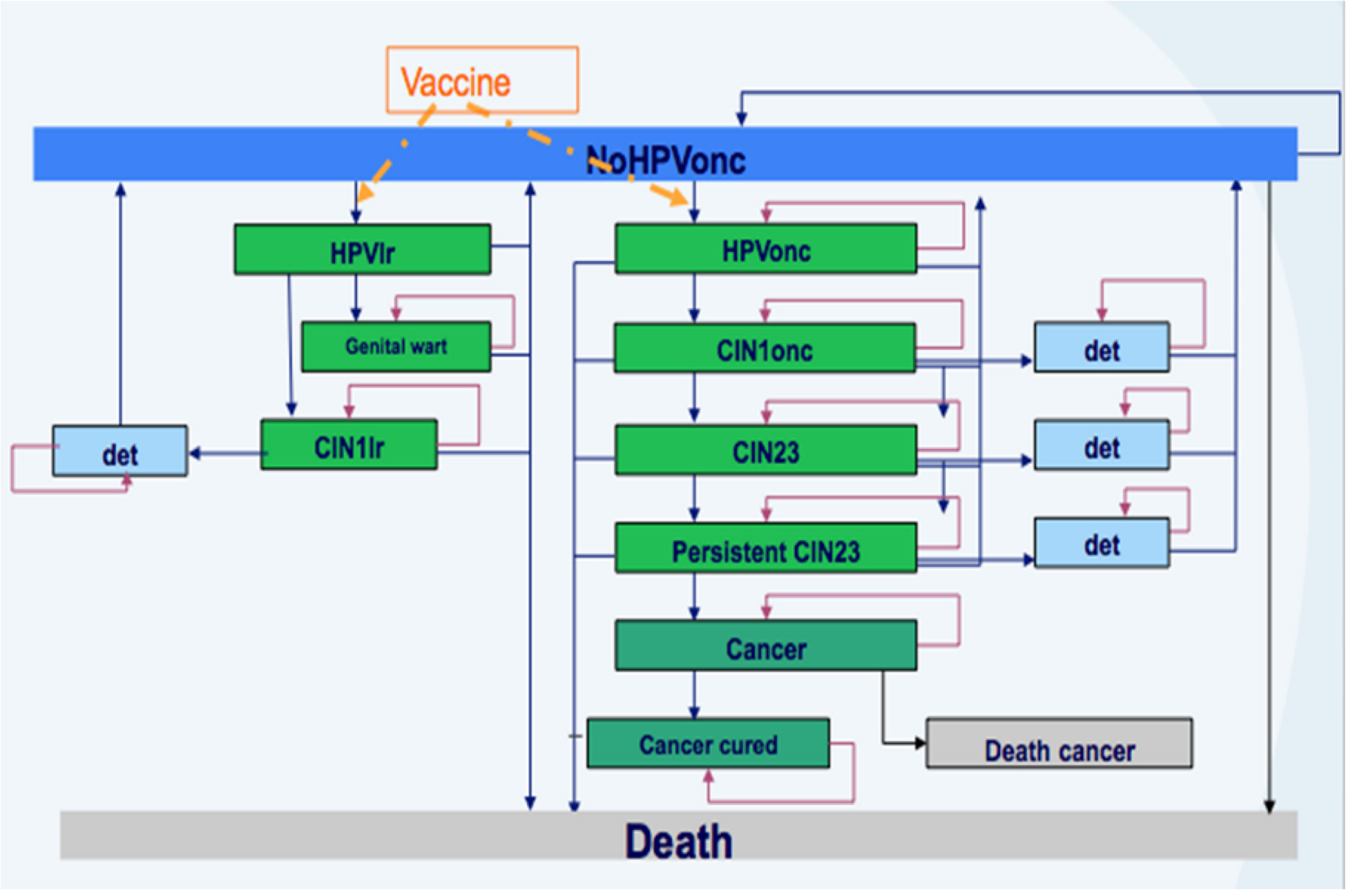
Markov Cohort Model structure

Cohorts enter the model in a health state free of oncogenic HPV (NoHPVOnc). With each cycle, there is probability of either remaining in that health state, or becoming infected with low risk HPV (HPVlr, on the left hand side) or oncogenic HPV (HPVonc, on the right hand side). In the next cycle, a subject infected with oncogenic HPV may remain in that health state, or move to oncogenic cervical intraepithelial neoplasia 1 (CIN1onc). Similarly, in each subsequent cycle, the subject may remain in the same health state or move one state closer to CC (from CIN1onc to CIN23 then Persistent CIN23 and finally Cancer). The Fig. 1 Model structure.

### Model setting

A Markov cohort model reflecting the natural history of HPV infection accounting for oncogenic and low-risk HPV was adapted for 13-year-old Malaysian girls cohort (*n* = 274,050) (http://www.statistics.gov.my/portal/index.php?lang=en). It was assumed that all Malaysian girls aged 13 years would receive the vaccination (i.e. 100 % vaccination coverage).

Subjects with CC will either be cured from cancer and thereafter die of natural causes, or may be deceased as a result of their CC. If an individual underwent screening and has an advanced disease stage detected through the screening, then she will be in the det health state which is different because of possibly more costly medical follow-up such that her risk of moving to a more advanced health state is reduced Movement between health states may take longer than one calendar year – for example, it may take more than 13 years to move from the first health state (NoHPV) to the last health state (Death CC).

There is a clear link between HPV virus infection and Cervical Cancer Screening early stages of the disease impacts the natural history of CC. Vaccination alters natural history of the disease at infection by impacting screening results as well as CC cases and deaths; thus it impacts all the pre-cancer stages. Based on the fundamental statements above, the model structure is designed as follows: Lifetime Markov cohort model simulating natural history of cervical cancer and incorporates the effects of screening and HPV vaccination, Model runs for 95 years to cover the total lifetime of the cohort, with each cycle being one year in duration, Cohort of 13 year old girls will be included, since that is the age of the school-based HPV vaccination program, Fixed transition probabilities determine cohort movement from one health state to the next in each cycle; each health state has an associated utility and cost which can then be combined with time spent in each state to estimate the total costs and QALY’s (subject to 3 % discount rate), Data related to the natural history of the disease are assumed constant across countries, Model is adapted to the Malaysian setting by incorporating local cost and epidemiological data inputs where available.

### Parameter values

Transition probabilities, utilities values, epidemiological data were sourced from published literature data [9, 10]. Vaccine effectiveness was based on overall efficacy reported from 3-doses clinical trials, with the assumption that the 2-doses is non-inferior to the 3 -doses allowing overall efficacy to be inferred from the 3-dosese immunogenicity data. Price parity and life-long protection were assumed [5, 11-13]. The costing data from local data, per dose of vaccine including administrative cost was RM 134, screening was RM27, CIN 1 diagnosis & treatment cost was RM1,905.41 , CIN 2/3 diagnosis & treatment cost was RM2,230.80 and Cancer treat costs (stages I-IV) was RM56,694.83. The payer perspective was adopted, with appropriate discounting for costs (3 %) and outcomes (3 %) based on Malaysian Pharmacoeconomic guidelines [14].

### Sensitivity analysis

One way sensitivity analysis was conducted. The sensitivity analysis on cost of vaccine by +/− 30 %, vaccine coverage by −20 and 40 %, Vaccine efficacy +/−2 % and discount rate by −1.5 % and +2 % with a 2D protocol was performed.

### Ethical approval

This study was approved by Ethics Committee of the Universiti Kebangsaan Malaysia (National University of Malaysia) Medical Centre (Code: FF-370-2012) and Ministry of Health Malaysia (Code: NMRR-12-1207-14109).

## Results

The Markov Cohort model predicted that both HPV vaccination schemes can avoid same number of CC 361 case, 126 Death and 7,732,266 QALY. In terms of the cost, RM36, 722,700 vaccine cost would be saved with 2-doses schedule vaccination compare to 3dose schedule. The model also predicted with 2 dose HPV vaccination scheme versus no vaccination programme, with the vaccination scheme can avoid more 1,237 CC cases, 428 deaths and 1,635 QALY compare to no vaccination programme. In terms of the cost, RM 9,827,304 would be saved with the 2 doses vaccination scheme. The result determined that 2-doses HPV vaccination scheme is cost saving compare to no vaccination and 3-doses. MOH Malaysia could vaccinate more girls at other age group (current vaccination with 3 dose schedule for 13 years old girls in the country) using saving money with 2 -doses schedule vaccination programme. With saving RM 36, 722, 700, MOH could vaccinated 137, 025 more girls in this country.

### Sensitivity analysis

One way sensitivity analysis was conducted. The sensitivity analysis on cost of vaccine, vaccine coverage and discount rate with a 2-doses protocol was performed.

#### Cost of vaccine

Current per dose of the vaccine cost is MYR 134. Hence, −30 % from the cost of the vaccine would results in a vaccine cost of MYR 94. Hence, when the cost is lowered by 30 %, the saving becomes MYR 25,760,700 (i.e. 274,050*94). A +30 % to vaccine cost would result in it becoming MYR 174. Thus, when the cost is raised by 30 %, the saving becomes MYR 47,684,700 (i.e. 274,050*174).

#### Vaccine coverage

Current coverage is 100 %, at 80 % coverage: the savings become MYR 29,378,160 (i.e. 274,050*0.8*134) and at 60 % coverage: the savings become MYR 22,033,620 i.e. 274,050*0.6*134)

#### Vaccine efficacy

Current vaccine effectiveness is 98 % on 16 and 18 types, when we increased the vaccine efficacy to 100 %, the QALYs gain becomes 1,663 while the vaccine efficacy decreased to 96 %, the saving QALYs gain slightly reduce to 1,607.

#### Discount rate

Current rate is 3 % for both costs and outcomes à QALYs saved is 6,012, at 1.5 %: QALYs saved becomes 23,998 and at 5 %: QALYs saved becomes 55,158.

## Discussion

The cost-effectiveness analysis was carried out using Markov cohort model to evaluate the health and economic impact of vaccinating 13-year-old girls in Malaysia with the no vaccination and 3-doses schedule vaccination versus 2-doses schedule and compared the outcomes of both strategies. The Markov model was therefore the most representative of the target group for universal mass vaccination. In the base case, it was assumed that the vaccine coverage was 100 % and that both vaccination schedules offered lifelong protection. 2-doses schedule would result in the prevention of same number of CC cases and number of death but in terms of cost, with 2-doses schedule could be saved RM 36,722,700, with this saving MOH could vaccinated 137,025 more girls in this country bases on this more population with 2-doses HPV vaccination schemes can avoid more 180 CC and 63 deaths compare to 3-doses. 2-doses schedule would prevent more number of CC cases, death and QALY but in terms of the cost, RM 9, 827,304 would be saved with the 2 dose vaccination scheme compare to no vaccination programme.

There are few limitations on this study first, unlike a dynamic model a cohort model cannot account for the benefits offered by herd effect, that is, protection of unvaccinated individuals by those vaccinated because of the reduced circulation of the infective agent [15-16]. Second, the model combines all oncogenic and low-risk HPV types together, and thus cannot account for the differential progression or regression for each HPV type. However, it provides a first estimation of the overall expected effect of both vaccines while limiting the number of input data for which no information is available.

## Conclusion

With 2-doses HPV vaccination regime may enable Malaysian women to be protected at a lower cost than that achievable under a 3-doses scheme, while avoiding at a population level the same number of Cervical Cancer cases and deaths. 2-doses scheme may ease budget constraints which would allow for future cohorts to be vaccinated as well.
